# Detemir plus aspart and glulisine induced lipoatrophy: 2015 literature review and report of a new case

**DOI:** 10.1186/s40842-015-0013-5

**Published:** 2015-10-15

**Authors:** Sima Saberi, Nazanene H. Esfandiari, Mark P. MacEachern, Meng H. Tan

**Affiliations:** 1 Ann Arbor Endocrinology and Diabetes, PC, Ypsilanti, Michigan USA; 2 Division of Metabolism, Endocrinology and Diabetes, University of Michigan, Lobby C, 24 Frank Lloyd Wright Drive, Ann Arbor, MI 48106 USA; 3 Taubman Health Sciences Library, University of Michigan, Ann Arbor, Michigan USA

**Keywords:** Lipoatrophy, Aspart, Detemir, Aspart plus detemir, NovoMix 30, Glulisine, Continuous subcutaneous insulin infusion

## Abstract

**Background:**

In the first and only literature review, conducted in 2009, of human insulin analog- induced lipoatrophy, there were 12 published cases, including 1 with aspart, 1 with detemir, 1 with NovoMix 30 and none with detemir plus aspart. It is perceived that insulin analog induced-lipoatrophy is increasing. We conducted a 2015 literature review of published reports of lipoatrophy induced by aspart, detemir, detemir plus aspart, and NovoMix30. We also report a new case of detemir plus aspart and glulisine induced lipoatrophy.

**Methods:**

Our focused literature searches (limited to 1995–2014) in PubMed, Embase, and Web of Science, using a combination of insulin analog and lipoatrophy terminology, was conducted in early January 2015.

**Results:**

From the 520 unique citations there were 33 (from 13 papers and 9 abstracts) lipoatrophy cases induced by detemir (n = 5), aspart (n = 21), detemir plus aspart (n = 4) and NovoMix 30 (n = 3), representing 30 new cases since 2009. Many of these reported cases were females (76 %), had type 1 diabetes mellitus (T1DM) (94 %) and were in young persons (61 %). A 41-year-old T1DM woman developed lipoatrophy on her upper thighs, arms and abdomen 14 months after injecting detemir plus aspart at the same sites. Later on, after a year on continuous subcutaneous insulin infusion (CSII) using aspart and then glulisine, she developed lipoatrophy at the infusion sites. When CSII insulin was switched to lispro she did not develop lipoatrophy after 10 months. Meanwhile, the original lipoatrophy sites significantly improved.

**Conclusions:**

Our literature review uncovered 30 new published cases of aspart, detemir, aspart plus detemir and NovoMix 30-induced lipoatrophy since 2009. The largest increase in cases was in aspart- induced lipoatrophy. Recent surveys showed most rapid acting insulin analog-induced lipoatrophy were associated with CSII. In our review of the reported cases, 85.7 % cases of aspart-induced lipoatrophy were associated with CSII. As in previous reports, we showed lipoatrophy was more common in females, T1DM and young persons. Our patient may be the 5^th^ published case of detemir plus aspart-induced lipoatrophy and possibly the first case report of glulisine induced lipoatrophy. She believed both detemir plus aspart and glulisine induced the lipoatrophy.

## Background

Insulin-induced lipoatrophy was a very common cutaneous complication of insulin therapy, found in 25- 55 % of patients injecting bovine and porcine insulin [1]. It became less common (<10 %) with purer animal and human insulin [1]; and uncommon (about 1 %) after human insulin analogs became available [2]. Lipoatrophy has been reported in patients using basal (glargine [3] and detemir [4]), rapid-acting (lispro [5], aspart [6] and glulisine [7]) and mixture [8] insulin analogs injections. In the first and only literature review of human insulin analog-induced lipoatrophy, done in November 2009 [9], there were 12 cases, including 1 with aspart, 1 with detemir, 1 with NovoMix 30 (Biphasic aspart – 30 % aspart 70 %, NPH insulin) and none with detemir plus aspart. Insulin analog induced lipoatrophy is perceived to be increasing in prevalence [2]. In this paper we report a literature review conducted in early January 2015 of published reports of lipoatrophy induced by aspart, detemir, detemir plus aspart, and NovoMix30 injections and report a new case of lipoatrophy induced by detemir plus aspart as well as glulisine injection. We will not cover lipoatrophy induced by lispro, lispro mixtures, and glargine in detail.

## Methods

We conducted focused literature searches in early January 2015 in PubMed, Embase, and Web of Science using a combination of insulin analog and lipoatrophy terminology. We used MeSH and EMTREE controlled terms when appropriate for broad concepts, such as “insulin analog” [mesh] and lipodystrophy [mesh:noexp]. We supplemented each controlled term with a comparable set of title/abstract keywords, going so far as to include all individual insulin analogs (‘lispro’, ‘humalog’, ‘aspart’, ‘novolog’, ‘detemir’, ‘levemir’, ‘glulisine’, ‘apidra’, and ‘glargine’, ‘lantus’). We also conducted a similar search in Web of Science to primarily identify conference abstracts that were not found in PubMed or Embase. We limited all searches to articles and abstracts published between 1995 (when the first insulin analog was launched) and 2014, and deliberately designed the searches to miss, when possible, citations pertaining to HIV-related lipoatrophy. We exported citations into Endnote X6 (Thomson Reuters) and used its functionality to eliminate duplicates.

We report a new case of a female type 1 diabetes mellitus (T1DM) patient who developed lipoatrophy when injecting detemir plus aspart in the same site. Later on, when using continuous subcutaneous insulin infusion (CSII) to deliver aspart and then glulisine, she also developed lipoatrophy at the infusion sites. We obtained written informed consent from the patient for publication of this case report and the images.

We requested information on lipoatrophy associated with aspart and/or detemir injections from the manufacturer of these insulin analogs.

## Results

### Literature search

**Fig. 1:**
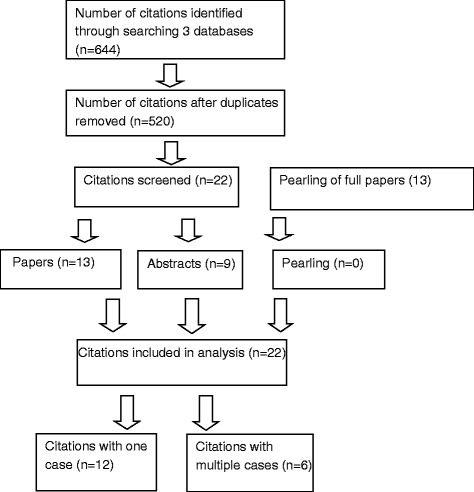
Flow chart showing the processes of identification, screening, elimination and inclusion in this review

The literature search of PubMed, EMBASE and Web of Science yielded 273, 252 and 119 citations respectively, giving a combined total of 644 citations (Fig. 1). After the duplicates were eliminated, we had 520 unique citations. Two authors (MT and NE) reviewed each of the 520 citations and concurred on those identified as lipoatrophy induced by insulin analog aspart, detemir, detemir plus aspart and NovoMix 30 injections. We found 33 reported cases from 18 citations (13 papers/letters/ observations/vignette and 9 abstracts). From the references of 13 papers, pearling was done to determine whether they included other published abstracts/papers on lipoatrophy induced by aspart, detemir, detemir plus aspart, NovoMix 30 and glulisine injections. We found none.

The 33 lipoatrophy cases are induced by detemir (n = 5) [4, 10, 11], aspart (n = 21) [6, 10, 12–22], detemir plus aspart (n = 4) [11, 23, 24], and NovoMix 30 (n = 3) [8, 10] injections (Table 1). One of the detemir cases [4], one of the aspart cases [6] and one of the NovoMix30 cases [8] were previously described in the 2009 literature review [9], giving 30 new cases since then: 4 detemir cases [10, 11], 20 aspart cases [10, 12–22], 2 NovoMix 30 [10], and 4 detemir plus aspart cases [11, 23, 24]. The characteristics of the 33 cases are:

**Table 1:** Thirty-three lipoatrophy cases induced by detemir, aspart, detemir plus aspart, and NovoMix30

Year published and country	Detemir	Aspart	Detemir plus aspart	NovoMix30 (30 % aspart plus 70 % NPH)	Glulisine
*Literature Search 2009 (9)*					
Hussein et al. UK 2007 [8]				65 yo Caucasian female with late-onset TIDM started on NovoMix 30. LA in both thighs (onset not mentioned). When switched to abdomen, new LA developed. Did not resolve.	
Szypowska et al. Poland 2008 [6]		32 mo Caucasian boy with T1DM using Asp in CSII. LA at infusion sites in buttocks 10 months later. Switched to Lispro and LA reported at new infusion sites.			
Del Olmo et al. Spain 2008 [4]	30 yo female T1DM. Detemir 10 u hs. LA appear on thighs “several” months after initiation. No LA with NPH, Mixtard 30 or Actrapid used earlier.				
*Literature Search 2015*					
Kesavadev J et al. India 2008 [12]		55 yo Indian male with T2DM (?) on CSII using Asp developed LA at infusion sites 2 months after initiating therapy.			
Bocca et al. Netherlands 2009 [13]		7 yo girl with T1DM since age 1 yr. Started on CSII using Asp at age 5 yrs. At age 7 yrs LA developed at infusion sites on buttocks and thighs.			
Chang YT et al. USA 2010 [14]		11 yo girl with T1DM on CSII using Asp. Developed LA at infusion sites after 1 yr. Previously on MDI using Asp + glargine with lipohypertrophy at injection sites. Switched to lispro and applied sodium cromoglicate cream with little effect.			
Ninnikoski et al. Finland 2010 [15]		7 yo boy with T1DM on CSII using Asp. Developed LA in infusion site on buttocks within a year of starting Asp. Switched to lispro and used a different site. Applied pimecrolimus cream with little effect on LA.			
Ninnikoski et al. Finland 2010 [15]		15 yo boy with T1DM on CSII using Asp. Developed LA in infusion site on thighs within a year of starting Asp. Switched to glulisine and changed site. LA did not disappear.			
Ninnikoski et al. Finland 2010 [15]		10 yo girl with T1DM since age 5 years on CSII using Asp. Developed LA in infusion sites on thighs 4 yrs later. Switched to lispro and changed site. Applied Na cromoglycate with some improvement in LA.			
Babiker et al. UK 2011 [10]				4 yo Caucasian T1DM NovoMix 30. LA 2-3 yrs after insulin. LA in new injection sites when site changed	
Babiker et al. UK 2011 [10]				5 yo Caucasian T1DM NovoMix 30. LA 3 yrs after insulin. LA in new injection sites with Lispro 25/75	
Babiker et al. UK 2011 [10]		12 yo Caucasian T1DM Novorapid+glargine. LA 3 yrs after insulin. Novorapid site only. Resolved when site changed			
Babiker et al. UK 2011 [10]	14 yo Caucasian T1DM Novorapid+detemir. LA 3 yrs after insulin. Detemir site only. LA when detemir changed to glargine.				
George PS et al. UK 2011 [16]		? yo woman with T1DM on CSII using Asp. Developed LA at infusion sites 2 years after initiation. Previously on MDI using glargine and Asp without LA.			
Yazdanyar S et al. Denmark 2011 [17]		17 yo girl with T1DM developed lipoatrophy at aspart infusion sites 1.5 years after starting CSII. Previously used Biphasic Asp. Switched to glulisine without lipoatrophy.			
Yazdanyar S et al. Denmark 2011 [17]		8 yo boy with T1DM developed LA as aspart infusion sites soon after starting CSII. Previously used aspart, Biphasic aspart and glulisine without LA.			
Yazdanyar S et al. Denmark 2011 [17]		7 yo girl with T1DM developed LA at Aspart infusion sites 10 months after starting CSII. Previously used Asp and Biphasic Asp without LA.			
Tavare AN et al. UK 2011 [23]			53 yo Indian woman with T2DM developed LA at Asp + Det injection sites. Previously on NovoMix 30 with itching and erythema. Also developed LA (onset not stated) and local reaction to Lispro, Lispro 25/75, glargine and Human Mixtard 30.		
Peteiro-Gonzalez D et al. Spain 2011 [18]		39 yo woman with T1DM on MDI-glargine and Asp. LA at Asp sites (thighs) 2 years after Asp therapy. Also had primary hypothyroidism and psoriasis. Biopsy and TNFα elevated.			
Salma et al. France 2011 [19]		42 yo man with T1DM since age 7 yrs. After CSII for 14 months, he developed LA. Applied Na Cromoglycate and switshed to glulisine.			
Swelheim et al. Netherlands 2012 [20]		7 yo female with T1DM CSII aspart. LA (few months later) in buttocks and thighs. Substituted with lispro with no benefit.			
Suththanantha J et al. UK 2012 [21]		7 yo girl with T1DM developed LA at Asp infusion site when on CSII. Onset not mentioned.			
Suththanantha et al. UK 2012 [21]		17 yo boy with T1DM developed LA at Asp injection sites (on MDI). Onset not mentioned. Had hypothyroidism and Addison’s disease.			
Agha et al. UK 2013 [11]	54 yo female with T1DM. Detemir in her thighs and Aspart in her abdomen. Lipoatrophy in her thighs. Hypoglycemia improved when injection site changed to abdomen.				
Agha et al. UK 2013 [11]	29 yo female with T1DM. Detemir in her thighs and Aspart in her abdomen. Lipoatrophy in her thighs after 2.5 years.				
Agha et al. UK 2013 [11]			26 yo T1DM woman on detemir and aspart developed LA at injection sites on her thighs. LA onset not mentioned		
Agha et al. UK 2013 [11]			32 yo T1DM woman on detemir plus aspart developed LA at injection sites on her thighs. LA onset not mentioned.		
Agha et al. UK 2013 [11]	64 yo female with T1DM. Detemir in her thighs for 8 years. LA in thighs 2-3 years after starting it.				
Breznik et al. Slovenia 2013 [24]			62 yo T2DM woman on detemir plus aspart developed LA 5.5 yrs after starting insulin. Did not resolve spontaneously. Biopsy findings		
Simeonovic M et al. UK and Australia 2014 [22]		3 yo girl with T1DM developed LA at Asp infusion site (buttock) when on CSII (after 1-3 yrs).			
Simeonovic M et al. UK and Australia 2014 [22]		7 yo girl with T1DM developed LA at Asp infusion site (abdomen) when on CSII (1-3 yrs later).			
Simeonovic M et al. UK and Australia 2014 [22]		8 yo girl with T1DM developed LA at Asp infusion site (thigh) when on CSII (after 1-3 yrs).			
Simeonovic M et al. UK and Australia 2014 [22]		10 yo girl with T1DM developed LA at Asp infusion site (abdomen) when on CSII (after 1-3 yrs).			
Saberi et al. USA 2014 (our report)			41 yo T1DM woman developed LA at injection sites on thighs, upper arms and abdomen 14 months after starting aspart and detemir MDI. When switched to CSII (Omnipod) using aspart LA developed within a year. When switched to CSII using glulisine LA developed after a year. When switched to CSII using lispro no new LA after 10 months. The original LA began to fill in when left alone.		41 yo T1DM woman developed LA at injection sites on thighs, upper arms and abdomen 14 months after starting aspart and detemir MDI. When switched to CSII (Omnipod) using aspart LA developed within a year. When switched to CSII using glulisine LA developed after a year. When switched to CSII using lispro no new LA after 10 months. The original LA began to fill in when left alone.

#### Gender

Of the 5 patients with detemir-induced lipoatrophy, the gender of 4 was stated. All were females. Of the 21 patients with aspart-induced lipoatrophy, the gender of 20 was stated. Of these, 13 were females and 7 were males. All 4 patients with detemir plus aspart-induced lipoatrophy were females. Of the 3 patients with NovoMix 30-induced lipoatrophy, 1 was female and the gender of the other 2 was not stated.

#### Type of diabetes

All 5 patients with detemir-induced lipoatrophy had T1DM. Twenty of the 21 patients with aspart-induced lipoatrophy had T1DM and one had type 2 diabetes mellitus (T2DM). Among the 4 adult female patients with detemir plus aspart-induced lipoatrophy, 2 had T1DM and 2 had T2DM. All 3 patients with NovoMix 30-induced lipoatrophy had T1DM.

#### Age

Four of the 5 patients with detemir-induced lipoatrophy were adults and 1 was an adolescent. Fourteen of the 21 patients with aspart-induced lipoatrophy were children, 3 were adults, 3 adolescents and 1 had no age mentioned. All 4 patients with detemir plus aspart- induced lipoatrophy were adults. Two patients with Novo-Mix 30-induced lipoatrophy were children and 1 an adult.

### Case

**Fig. 2:**
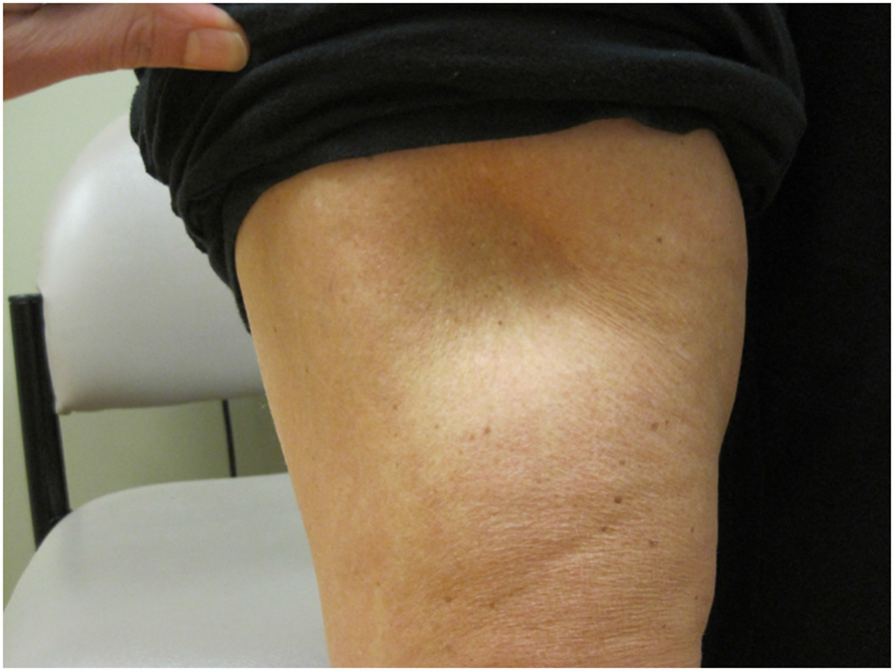
Lipoatrophy on thigh in November 2012

**Fig. 3:**
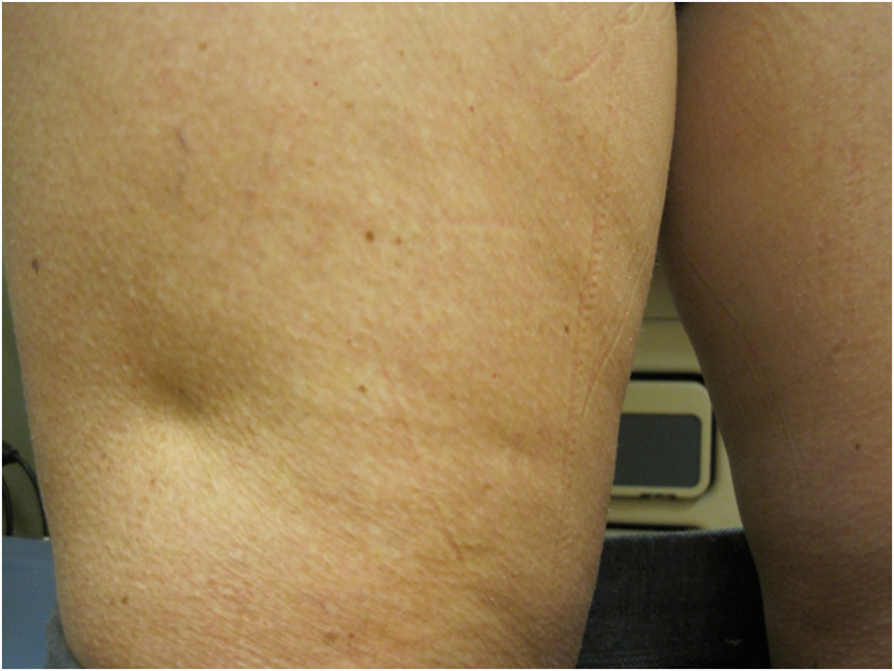
Improved lipoatrophy on thigh in December 2014

We report a 41-year-old Caucasian woman with T1DM diagnosed in April 2010 who developed lipoatrophy on her upper thighs, arms and abdomen injection sites in June 2011 after being on multiple daily insulin regimen of detemir plus aspart for 14 months (Fig. 2). Her Hemoglobin A1c improved from 10.9 % at diagnosis to 6.4-7.4 % after starting these insulin analogs. She injected both insulin analogs in the same sites and could not specify which insulin analog was the cause of these indentations. In September 2011 she started CSII using Omnipod with aspart. New lipoatrophy developed at the infusion sites within a year. In November 2012 glulisine was used instead of aspart. When the infusion was above or below the lipoatrophy thigh sites, the lipoatrophy worsened. Therefore, she switched to abdominal sites and developed new lipoatrophy there. She was then evaluated by allergists at 2 different tertiary centers. One recommended steroid therapy and the other recommended observation. She chose clinical observation without steroid treatment. In February 2014 lispro was used instead of glulisine because the original lipoatrophy sites were not improving. In December 2014, 10 months after switching to lispro and using abdominal sites only, no new lipoatrophy had appeared at infusion sites. Her latest A1C in December 2014 was 7.2 %. Meanwhile the original lipoatrophy sites in the upper thighs, upper arms and abdomen improved significantly (Fig. 3).

### Information from the manufacturer

In their response to our request for information on lipoatrophy induced by detemir, aspart, detemir plus aspart the manufacturer stated they did not have incidence data on lipoatrophy associated with these insulin analogs [25].

### Lipoatrophy induced by other insulin analogs

In the same literature search 17 cases of lispro, 2 cases of lispro mixture, and 7 cases of glargine induced lipoatrophy were reported. No reported case of glulisine-induced lipoatrophy was found. These cases are not described in detail in this paper; but their details can be obtained from the authors. Our new case of detemir plus aspart induced lipoatrophy also developed lipoatrophy when using glulisine, making it the first published reported case of glulisine induced lipoatrophy.

## Discussion

Based on our 2015 literature search of published reports, our adult female patient with T1DM may be the 5^th^ published case of detemir plus aspart-induced lipoatrophy. Aspart was launched in 2000 and detemir in 2006. The first published report on detemir plus aspart-induced lipoatrophy in 2011 was followed by 3 more cases in 2013. Among the previously reported 4 adult female patients with detemir plus aspart-induced lipoatrophy, 2 had T1DM [11] and 2 had T2DM [23, 24]. When both detemir and aspart are injected in the same site, it is difficult to identify whether one or both of these insulin analogs induced the lipoatrophy as either can cause it. According to our patient, she injected aspart and detemir in the same sites and could not determine which insulin analog induced the lipoatrophy. When she was on CSII using aspart and then glulisine she also developed lipoatrophy at the infusion sites.

In the past, CSII was reported to treat lipoatrophy induced by human insulin [26]. Today, lipoatrophy induced by rapid-acting insulin analog is often associated with CSII. Two recent surveys reported 83.3 % [27] and 87 % [2] of patients with lipoatrophy induced by rapid-acting insulin analogs use CSII. In our report 18 of the 21 (85.7 %) cases of aspart-induced lipoatrophy were associated with CSII and only 3 cases injected aspart. The infusion may cause the lipoatrophy [22]. Together continuous exposure to insulin and continuous mechanical irritation by the infusion catheter may trigger events that lead to lipoatrophy. Some patients did not develop lipoatrophy when injecting aspart but did so when infusing aspart via CSII [17, 22]. Others develop lipoatrophy with both injection and infusion implying the delivery method does not matter. Our patient had aspart-induced lipoatrophy within a year at the infusion sites when on CSII. She also developed lipoatrophy at the infusion sites when infusing glulisine via CSII after a year. Ten months after infusing lispro via CSII, she has not developed lipoatrophy at the infusion sites. This may be because she has not infused lispro long enough (one year or longer) as lipoatrophy can develop from 4 weeks [9] to 5.5 years [24] after starting insulin. It may also be because lispro does not induce lipoatrophy in her. Although all 5 insulin analogs can induce lipoatrophy, some patients have lipoatrophy induced by one but not another [17].

### Clinical presentation

Lipoatrophy is the loss of subcutaneous fat at insulin injection/infusion sites as demonstrated by biopsy [18, 28] and MRI [29]. In the past, 10 - 55 % of diabetic patients injecting impure bovine or porcine insulin developed lipoatrophy [1, 30]. With the availability of purer animal and human insulin, the prevalence of lipoatrophy decreased to 0.2-1.4 % [30, 31]. With insulin analog injections/infusions, the prevalence of lipoatrophy had been reported to be 2.5 % in a single center study [27] and 1.1 % in a multicenter survey [2].

There are no reported objective data for exact onset of insulin-induced lipoatrophy. The reported first observed onsets of insulin-induced lipoatrophy range from 4 weeks to 2 years [9], 2–3 to 23 months [26] to 6–24 months [1] after initiation of insulin therapy. In our literature search the first observed onsets of lipoatrophy range from 2 months to 5.5 years.

Lipoatrophy is more common in females in reported cases of lipoatrophy. In our review, the gender of 29 patients was identified. Of these 22 (75.8 %) were females and 7 males. Our patient is female. Why lipoatrophy is more prevalent in women remains unclear. There may be a reporting bias in this female gender predominance as this data is based on case reports and not clinical trials or MedWatch reports.

Lipoatrophy can overlap with other autoimmune diseases [21, 32]. Female T1DM patients with lipoatrophy have a higher risk of developing Hashimoto’s thyroiditis and celiac disease [32]. Autoimmune diseases affect 8 % of the population and of these 78 % are females [33]. Whether lipoatrophy and autoimmune diseases in females share a common etiology remains to be established. In females with T1DM and insulin-induced lipoatrophy, the physician should screen for other autoimmune disease(s). Our patient has Hashimoto’s thyroiditis with hypothyroidism; she does not have celiac disease or primary adrenal insufficiency.

Many of the reported cases of lipoatrophy induced by insulin analogs are in the pediatric population. Why this is remains unanswered. In our review, 20 (60.6 %) of the 33 cases were in the pediatric age group: 1 with detemir, 17 with aspart, and 2 with NovoMix 30. Like the previous 4 reported cases of lipoatrophy induced by detemir plus aspart injections, our case is an adult. Similarly, 4 of the 5 cases of lipoatrophy induced by detemir were adults. This probably reflects the age of patients using detemir plus aspart and detemir alone. Lipoatrophy more commonly occurs in T1DM partly because many cases are in the pediatric age group and possibly due to a potential immune-mediated inflammation causing lipoatrophy in patients with autoimmune type 1 diabetes. In our report, 31 of the 33 patients had T1DM and 2 had T2DM. Our patient has T1DM.

Lipohypertrophy sites from insulin injection can lead to impaired insulin absorption [34, 35]. To the best of our knowledge there are no published insulin absorption studies done in insulin analog-induced lipoatrophy sites. But, it is reasonable to expect variable insulin absorption in lipoatrophy sites with loss of adipose tissue, making glycemic control challenging. Some patients improved their glycemic control when insulin injection site is changed from the lipohypertrophy to an unaffected site [36]. One patient with lipoatrophy had improved glycemic control when the injection site was changed [11]. Another patient improved his glycemic control when given intra-peritoneal insulin suggesting that the lipoatrophy may have been causing poor insulin absorption [37]. Changing the injection or cannula site in our patient did not significantly affect her glycemic control. Clinically, insulin-induced lipoatrophy can be cosmetically distressing and disfiguring to the patient. For these reasons, the clinician should inspect the insulin injection/infusion sites regularly to identify lipodystrophy (lipohypertrophy and/or lipoatrophy), especially in patients with erratic glycemic control, so measures to manage the problem and prevent the development of further areas of lipoatrophy can be implemented.

As all these are reported cases, the number of cases of lipoatrophy induced by each insulin analog cannot be compared. However, all insulin analogs can induce lipoatrophy.

### Etiology

Many possible etiologies have been considered for insulin-induced lipoatrophy. These include cresol preservative in insulin, alcohol used for sterilization of syringes and needles, injury to the fat cells, glycolytic ferments in the insulin, possible nerve injury [38], mechanical trauma from repeat injections, and cryotrauma from cold insulin [10]. Immune etiologies have also been suggested - immune reaction to insulin [39, 40] and immune complex mediated inflammation [26]. Lipoatrophic lesions occurred in individuals using animal insulins who have high levels of circulating anti-insulin antibodies and the edges of lipoatrophy lesions were characterized by deposition of immunologic proteins within dermal vessel [39]. Although this had been questioned, a strong relationship between lipoatrophy and insulin antibodies was recently reported in adults with T2DM on recombinant human insulin or insulin analogs [41]. The immune-complex mediated inflammatory response involves local macrophage release of tumor necrosis factor α causing adipocyte dedifferentiation [26, 42] in patients using both animal and recombinant human insulin. Increased numbers of degranulating mast cells that stain positively for tryptase and chymase antibodies have also been seen in skin biopsies of patients with lipoatrophic sites of insulin analogs [43]. Histology has shown small adipocyte lobules with hyperplastic capillaries, loss of adipose tissue, areas of membranous lipodystrophy usually lined by an acellular homogeneous eosinophilic material and a focal lymphoid cell infiltration abutting hypodermis blood vessels [26, 44] in patients using both animal and human insulins. Although several studies have demonstrated a possible immune basis to the etiology of lipoatrophy, there are others which do not. Jermendy et al. reported absence of inflammatory cells and no local immune mechanisms in the biopsy specimen [44]. Milan et al. suggested that adipose tissue metabolic changes play a role in lipoatrophy as they did not identify any inflammatory cells in the skin biopsy specimens of their three T1DM patients with lipoatrophy by insulin analogs [28]. This study demonstrated a decrease in fat cell volume with adipocytes losing their lipid content leading to the hypothesis that adipocytes chronically exposed to high local insulin levels could develop insulin resistance resulting in an increase in the lipolytic process causing lipoatrophy. A significant down-regulation of leptin expression along with an increase in free fatty acid was also seen which was thought to result in the recruitment of fat cell precursors [28].

### Treatment

A change in insulin formulation, avoiding injections in the lipoatrophy sites (our patient noted this when the Omnipod was used near the sites), and changing the insulin needle daily have helped resolve lipoatrophy in some patients [3]. There are also case reports of adding glucocorticoid therapy such as dexamethasone or betamethasone to the insulin analog [20, 45]; but this can cause blood glucose fluctuations if the betamethasone/insulin analog solution becomes inhomogenous resulting in erratic insulin administration [20]. Administering low-dose oral glucocorticoid such as prednisone 5–10 mg daily [46, 47] has also been used to improve lipoatrophy. Corticosteroids are able to induce differentiation of adipocytes and have immune-modulating properties [45]. However, addition of glucocorticoid therapy can result in worsening glycemic control and increased insulin requirements [20]. Our patient declined the recommendation of using steroids. Yet, with time and not using the lipoatrophy sites, the original lipoatrophy began to improve (Fig. 3).

Changing the mode of insulin delivery, such as using CSII, can potentially improve lipoatrophy in patients using human insulin injections [48]. However, in our literature review, 18 of the 21 cases of lipoatrophy induced by aspart were associated with CSII which has been hypothesized to contribute to the development of lipoatrophy [22]. Why the largest increase in aspart-induced lipoatrophy occurred in those on CSII remains unclear. There may be a reporting bias in this group as this data is based on case reports and not clinical trials and Medwatch reports. Our patient developed lipoatrophy when on CSII using aspart and glulisine.

In 2 small studies topical 4 % sodium cromolyn in petrolatum solvent was partially effective therapy for early lipoatrophy areas and prevention of the development of new such areas [43, 49]. Our literature review described 2 other cases of lipoatrophy induced by aspart which improved with sodium cromolyn therapy [15, 19]. Cromolyn stabilizes mast cells that are tryptase-positive/chymase-positive and prevents the release of histamine in the presence of antigen-IgE antibody reactions [43].

Finally, 2 case reports described treatment of insulin-induced lipoatrophy. The first one is treating lipoatrophy successfully with an insulin jet –injector [50]. In an extremely refractory case Noud et al. used intraperitoneal insulin delivered by Diaport [37]. Inhaled insulin can potentially be used in patients with lipoatrophy induced by injected insulin. Lipoatrophy was not described as an adverse event in the Afreeza® product monograph [51]. We are aware that in Afreeza® trials high anti-insulin antibodies titers were documented. To the best of our knowledge no case of insulin lipoatrophy has been described in the Afreeza® clinical trials. Possible explanations for this include [a] without repeated injections of insulin no atrophy occurs despite the high insulin antibodies tiers; [b] an uncommon complication like insulin induced lipoatrophy may not appear in the limited number of patients who have used Afreeza thus far; and [c] the association of high insulin antibodies titers and injected insulin lipoatrophy does not imply a cause-effect association.

## Conclusion

Our case may be the 5^th^ case of detemir plus aspart induced lipoatrophy and possibly the first published case report of glulisine induced lipoatrophy. In prescribing information for Apidra [7] lipoatrophy was mentioned. There are 30 new published cases of aspart, detemir, detemir plus aspart and NovoMix induced lipoatrophy since 2009. These represent only a percentage of the cases of lipoatrophy induced by these insulin analogs. The ISPAD survey [2], the single center study [27] and the recent Rosenbloom recidivus [38] mentioned many cases (some due to these insulin analogs) which have not been published. To better understand insulin analog-induced lipoatrophy, more research on the prevalence of this cutaneous complication of insulin therapy, its etiology, pathogenesis and management need to be conducted. It is unrealistic to expect every case of insulin analog induced lipoatrophy to be published. But, sharing of data on reported, but not published, cases can be helpful.
